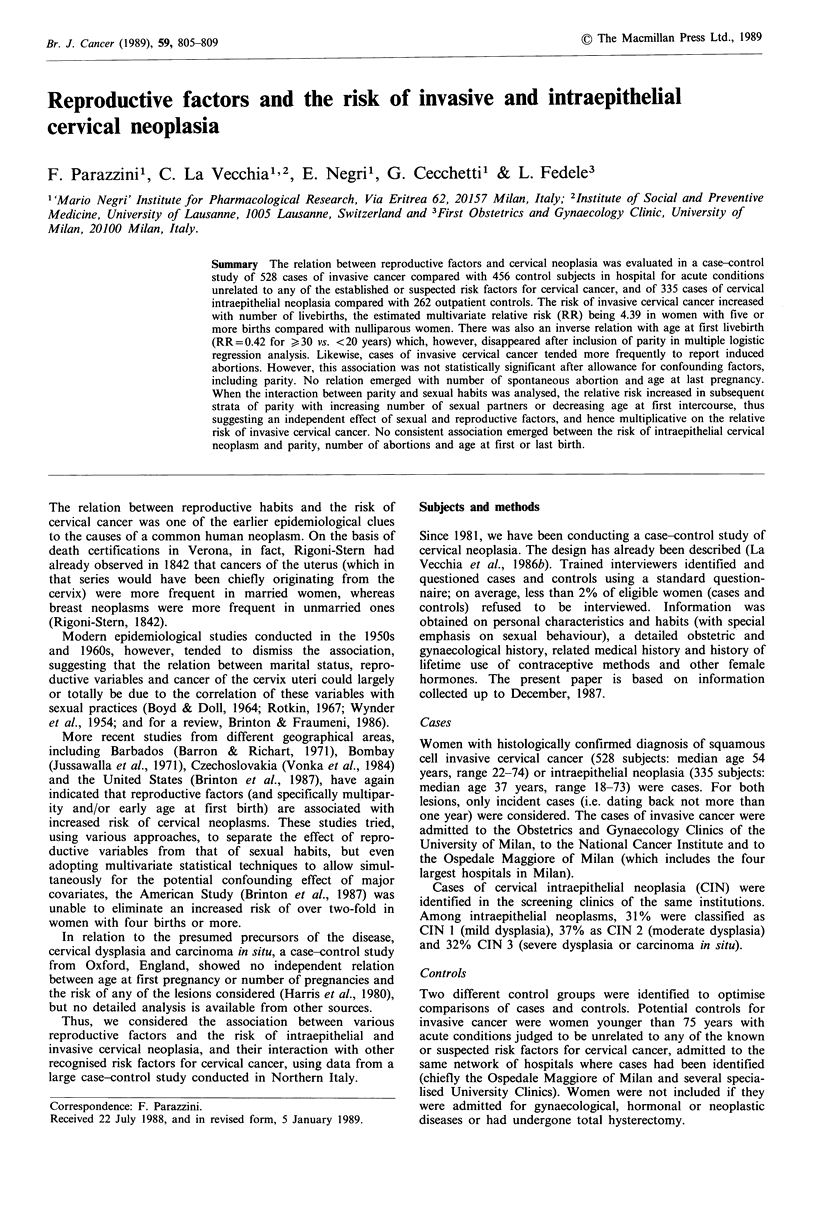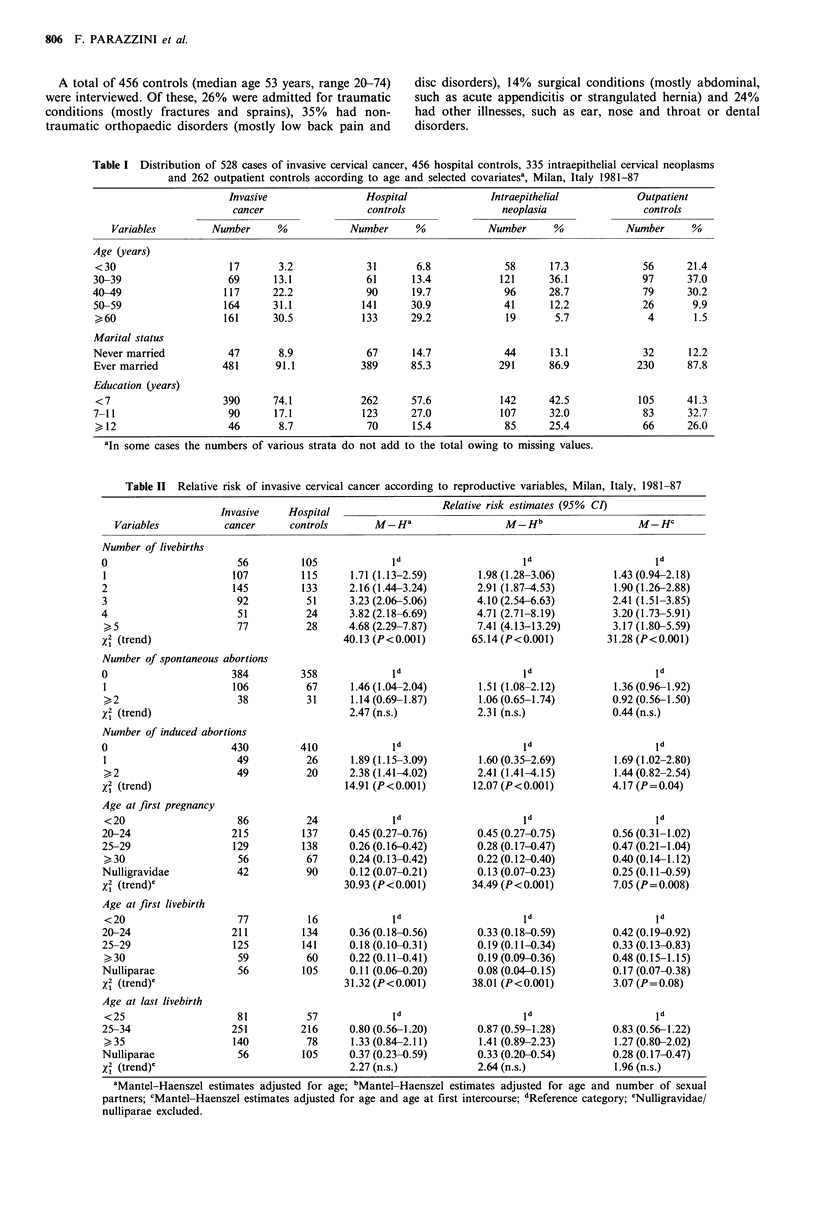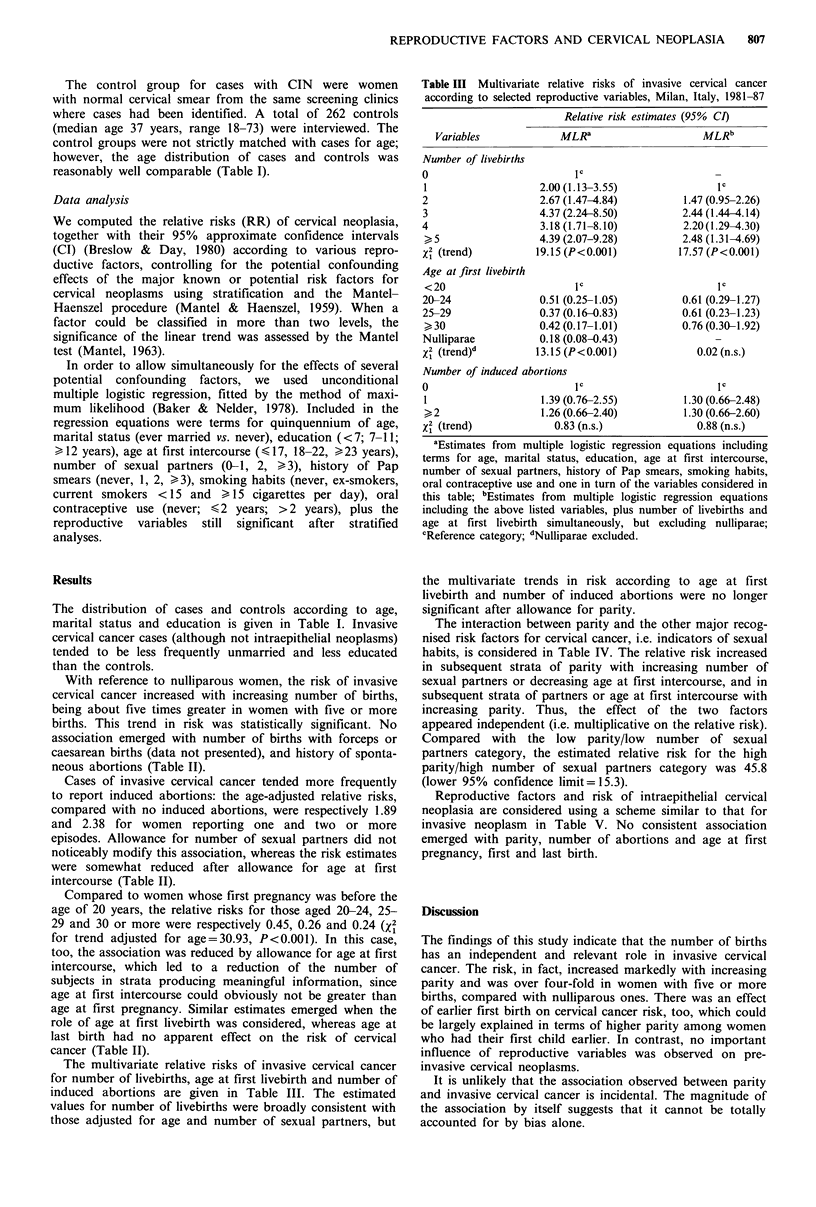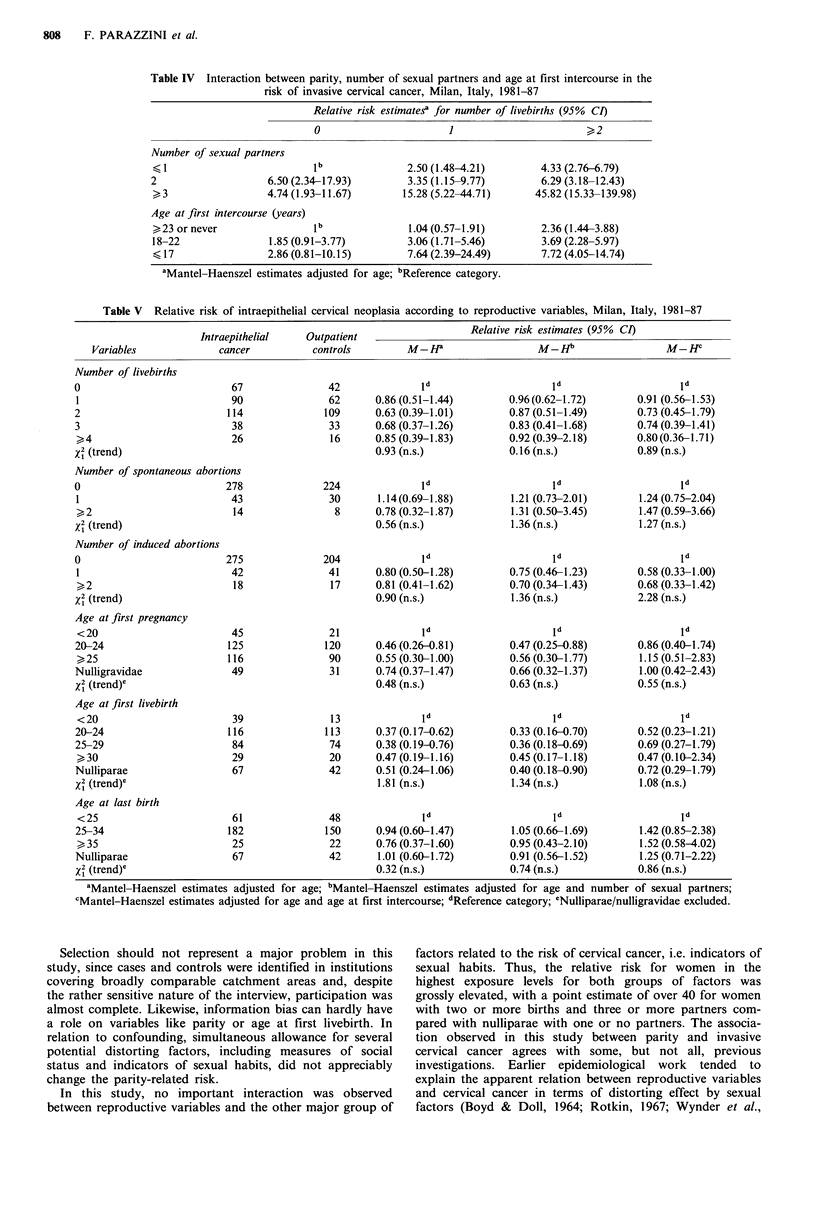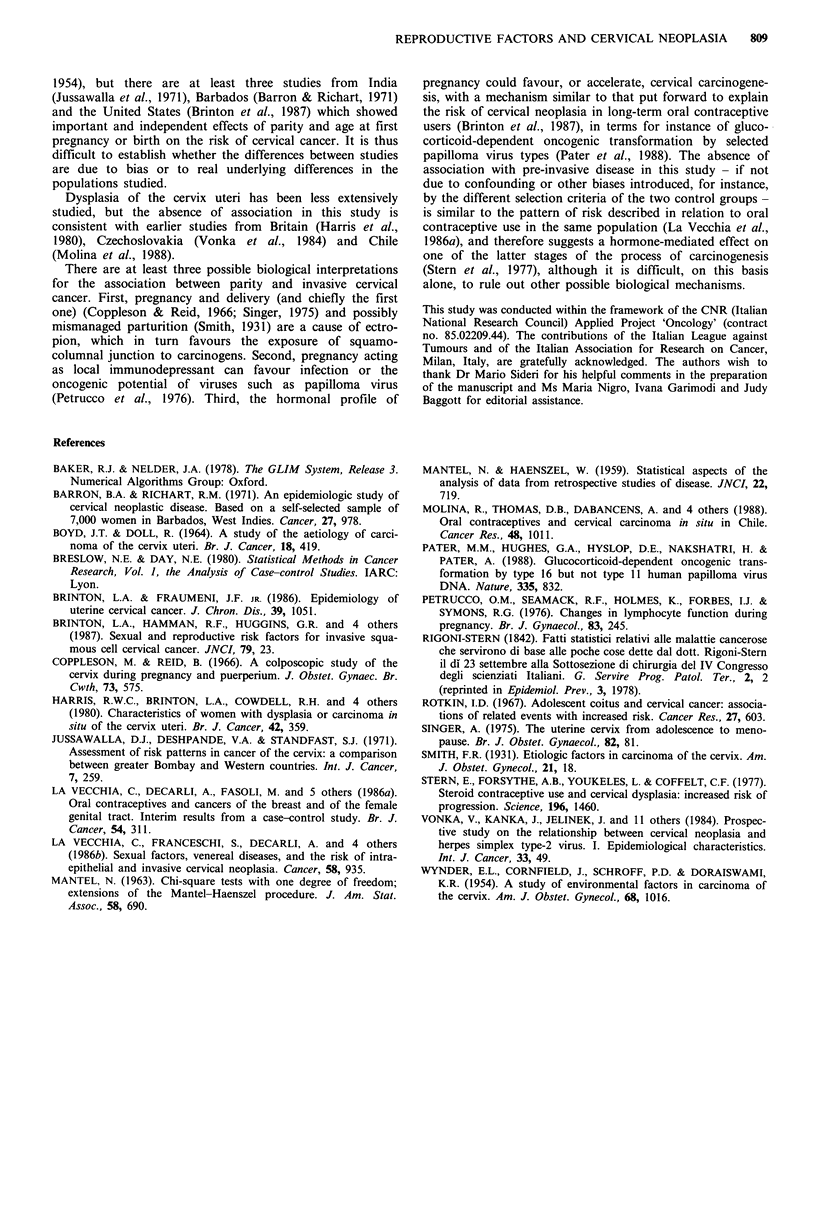# Reproductive factors and the risk of invasive and intraepithelial cervical neoplasia.

**DOI:** 10.1038/bjc.1989.168

**Published:** 1989-05

**Authors:** F. Parazzini, C. La Vecchia, E. Negri, G. Cecchetti, L. Fedele

**Affiliations:** Mario Negri Institute for Pharmacological Research, Milan, Italy.

## Abstract

The relation between reproductive factors and cervical neoplasia was evaluated in a case-control study of 528 cases of invasive cancer compared with 456 control subjects in hospital for acute conditions unrelated to any of the established or suspected risk factors for cervical cancer, and of 335 cases of cervical intraepithelial neoplasia compared with 262 outpatient controls. The risk of invasive cervical cancer increased with number of livebirths, the estimated multivariate relative risk (RR) being 4.39 in women with five or more births compared with nulliparous women. There was also an inverse relation with age at first livebirth (RR = 0.42 for greater than or equal to 30 vs. less than 20 years) which, however, disappeared after inclusion of parity in multiple logistic regression analysis. Likewise, cases of invasive cervical cancer tended more frequently to report induced abortions. However, this association was not statistically significant after allowance for confounding factors, including parity. No relation emerged with number of spontaneous abortion and age at last pregnancy. When the interaction between parity and sexual habits was analysed, the relative risk increased in subsequent strata of parity with increasing number of sexual partners or decreasing age at first intercourse, thus suggesting an independent effect of sexual and reproductive factors, and hence multiplicative on the relative risk of invasive cervical cancer. No consistent association emerged between the risk of intraepithelial cervical neoplasm and parity, number of abortions and age at first or last birth.


Br. J. Cancer (1989), 59, 805–809

© The Macmillan Press Ltd., 1989

### Reproductive factors and the risk of invasive and intraepithelial cervical neoplasia

F. Parazzini[1], C. La Vecchia[1,2], E. Negri[1], G. Cecchetti[1] & L. Fedele[3]

[1]*'Mario Negri' Institute for Pharmacological Research, Via Eritrea 62, 20157 Milan, Italy;* [2]*Institute of Social and Preventive Medicine, University of Lausanne, 1005 Lausanne, Switzerland and* [3]*First Obstetrics and Gynaecology Clinic, University of Milan, 20100 Milan, Italy.*

**Summary** The relation between reproductive factors and cervical neoplasia was evaluated in a case–control study of 528 cases of invasive cancer compared with 456 control subjects in hospital for acute conditions unrelated to any of the established or suspected risk factors for cervical cancer, and of 335 cases of cervical intraepithelial neoplasia compared with 262 outpatient controls. The risk of invasive cervical cancer increased with number of livebirths, the estimated multivariate relative risk (RR) being 4.39 in women with five or more births compared with nulliparous women. There was also an inverse relation with age at first livebirth (RR=0.42 for ⩾30 *vs.* <20 years) which, however, disappeared after inclusion of parity in multiple logistic regression analysis. Likewise, cases of invasive cervical cancer tended more frequently to report induced abortions. However, this association was not statistically significant after allowance for confounding factors, including parity. No relation emerged with number of spontaneous abortion and age at last pregnancy. When the interaction between parity and sexual habits was analysed, the relative risk increased in subsequent strata of parity with increasing number of sexual partners or decreasing age at first intercourse, thus suggesting an independent effect of sexual and reproductive factors, and hence multiplicative on the relative risk of invasive cervical cancer. No consistent association emerged between the risk of intraepithelial cervical neoplasm and parity, number of abortions and age at first or last birth.

The relation between reproductive habits and the risk of cervical cancer was one of the earlier epidemiological clues to the causes of a common human neoplasm. On the basis of death certifications in Verona, in fact, Rigoni-Stern had already observed in 1842 that cancers of the uterus (which in that series would have been chiefly originating from the cervix) were more frequent in married women, whereas breast neoplasms were more frequent in unmarried ones (Rigoni-Stern, 1842).

Modern epidemiological studies conducted in the 1950s and 1960s, however, tended to dismiss the association, suggesting that the relation between marital status, reproductive variables and cancer of the cervix uteri could largely or totally be due to the correlation of these variables with sexual practices (Boyd & Doll, 1964; Rotkin, 1967; Wynder *et al.*, 1954; and for a review, Brinton & Fraumeni, 1986).

More recent studies from different geographical areas, including Barbados (Barron & Richart, 1971), Bombay (Jussawalla *et al.*, 1971), Czechoslovakia (Vonka *et al.*, 1984) and the United States (Brinton *et al.*, 1987), have again indicated that reproductive factors (and specifically multiparity and/or early age at first birth) are associated with increased risk of cervical neoplasms. These studies tried, using various approaches, to separate the effect of reproductive variables from that of sexual habits, but even adopting multivariate statistical techniques to allow simultaneously for the potential confounding effect of major covariates, the American Study (Brinton *et al.*, 1987) was unable to eliminate an increased risk of over two-fold in women with four births or more.

In relation to the presumed precursors of the disease, cervical dysplasia and carcinoma *in situ*, a case–control study from Oxford, England, showed no independent relation between age at first pregnancy or number of pregnancies and the risk of any of the lesions considered (Harris *et al.*, 1980), but no detailed analysis is available from other sources.

Thus, we considered the association between various reproductive factors and the risk of intraepithelial and invasive cervical neoplasia, and their interaction with other recognised risk factors for cervical cancer, using data from a large case–control study conducted in Northern Italy.

Correspondence: F. Parazzini.
Received 22 July 1988, and in revised form, 5 January 1989.

### Subjects and methods

Since 1981, we have been conducting a case–control study of cervical neoplasia. The design has already been described (La Vecchia *et al.*, 1986*b*). Trained interviewers identified and questioned cases and controls using a standard questionnaire; on average, less than 2% of eligible women (cases and controls) refused to be interviewed. Information was obtained on personal characteristics and habits (with special emphasis on sexual behaviour), a detailed obstetric and gynaecological history, related medical history and history of lifetime use of contraceptive methods and other female hormones. The present paper is based on information collected up to December, 1987.

### *Cases*

Women with histologically confirmed diagnosis of squamous cell invasive cervical cancer (528 subjects: median age 54 years, range 22–74) or intraepithelial neoplasia (335 subjects: median age 37 years, range 18–73) were cases. For both lesions, only incident cases (i.e. dating back not more than one year) were considered. The cases of invasive cancer were admitted to the Obstetrics and Gynaecology Clinics of the University of Milan, to the National Cancer Institute and to the Ospedale Maggiore of Milan (which includes the four largest hospitals in Milan).

Cases of cervical intraepithelial neoplasia (CIN) were identified in the screening clinics of the same institutions. Among intraepithelial neoplasms, 31% were classified as CIN 1 (mild dysplasia), 37% as CIN 2 (moderate dysplasia) and 32% CIN 3 (severe dysplasia or carcinoma *in situ*).

### *Controls*

Two different control groups were identified to optimise comparisons of cases and controls. Potential controls for invasive cancer were women younger than 75 years with acute conditions judged to be unrelated to any of the known or suspected risk factors for cervical cancer, admitted to the same network of hospitals where cases had been identified (chiefly the Ospedale Maggiore of Milan and several specialised University Clinics). Women were not included if they were admitted for gynaecological, hormonal or neoplastic diseases or had undergone total hysterectomy.

A total of 456 controls (median age 53 years, range 20–74) were interviewed. Of these, 26% were admitted for traumatic conditions (mostly fractures and sprains), 35% had non-traumatic orthopaedic disorders (mostly low back pain and disc disorders), 14% surgical conditions (mostly abdominal, such as acute appendicitis or strangulated hernia) and 24% had other illnesses, such as ear, nose and throat or dental disorders.

**Table I** Distribution of 528 cases of invasive cervical cancer, 456 hospital controls, 335 intraepithelial cervical neoplasms and 262 outpatient controls according to age and selected covariates[a], Milan, Italy 1981–87

| | *Invasive cancer* | | *Hospital controls* | | *Intraepithelial neoplasia* | | *Outpatient controls* | |
|---|---|---|---|---|---|---|---|---|
| *Variables* | *Number* | *%* | *Number* | *%* | *Number* | *%* | *Number* | *%* |
| *Age (years)* | | | | | | | | |
| <30 | 17 | 3.2 | 31 | 6.8 | 58 | 17.3 | 56 | 21.4 |
| 30–39 | 69 | 13.1 | 61 | 13.4 | 121 | 36.1 | 97 | 37.0 |
| 40–49 | 117 | 22.2 | 90 | 19.7 | 96 | 28.7 | 79 | 30.2 |
| 50–59 | 164 | 31.1 | 141 | 30.9 | 41 | 12.2 | 26 | 9.9 |
| ⩾60 | 161 | 30.5 | 133 | 29.2 | 19 | 5.7 | 4 | 1.5 |
| *Marital status* | | | | | | | | |
| Never married | 47 | 8.9 | 67 | 14.7 | 44 | 13.1 | 32 | 12.2 |
| Ever married | 481 | 91.1 | 389 | 85.3 | 291 | 86.9 | 230 | 87.8 |
| *Education (years)* | | | | | | | | |
| <7 | 390 | 74.1 | 262 | 57.6 | 142 | 42.5 | 105 | 41.3 |
| 7–11 | 90 | 17.1 | 123 | 27.0 | 107 | 32.0 | 83 | 32.7 |
| ⩾12 | 46 | 8.7 | 70 | 15.4 | 85 | 25.4 | 66 | 26.0 |

[a]In some cases the numbers of various strata do not add to the total owing to missing values.

**Table II** Relative risk of invasive cervical cancer according to reproductive variables, Milan, Italy, 1981–87

| *Variables* | *Invasive cancer* | *Hospital controls* | *Relative risk estimates (95% CI)* $M-H^a$ | $M-H^b$ | $M-H^c$ |
|---|---|---|---|---|---|
| *Number of livebirths* | | | | | |
| 0 | 56 | 105 | 1[d] | 1[d] | 1[d] |
| 1 | 107 | 115 | 1.71 (1.13–2.59) | 1.98 (1.28–3.06) | 1.43 (0.94–2.18) |
| 2 | 145 | 133 | 2.16 (1.44–3.24) | 2.91 (1.87–4.53) | 1.90 (1.26–2.88) |
| 3 | 92 | 51 | 3.23 (2.06–5.06) | 4.10 (2.54–6.63) | 2.41 (1.51–3.85) |
| 4 | 51 | 24 | 3.82 (2.18–6.69) | 4.71 (2.71–8.19) | 3.20 (1.73–5.91) |
| ⩾5 | 77 | 28 | 4.68 (2.29–7.87) | 7.41 (4.13–13.29) | 3.17 (1.80–5.59) |
| $\chi^2_1$ (trend) | | | 40.13 ($P<0.001$) | 65.14 ($P<0.001$) | 31.28 ($P<0.001$) |
| *Number of spontaneous abortions* | | | | | |
| 0 | 384 | 358 | 1[d] | 1[d] | 1[d] |
| 1 | 106 | 67 | 1.46 (1.04–2.04) | 1.51 (1.08–2.12) | 1.36 (0.96–1.92) |
| ⩾2 | 38 | 31 | 1.14 (0.69–1.87) | 1.06 (0.65–1.74) | 0.92 (0.56–1.50) |
| $\chi^2_1$ (trend) | | | 2.47 (n.s.) | 2.31 (n.s.) | 0.44 (n.s.) |
| *Number of induced abortions* | | | | | |
| 0 | 430 | 410 | 1[d] | 1[d] | 1[d] |
| 1 | 49 | 26 | 1.89 (1.15–3.09) | 1.60 (0.35–2.69) | 1.69 (1.02–2.80) |
| ⩾2 | 49 | 20 | 2.38 (1.41–4.02) | 2.41 (1.41–4.15) | 1.44 (0.82–2.54) |
| $\chi^2_1$ (trend) | | | 14.91 ($P<0.001$) | 12.07 ($P<0.001$) | 4.17 ($P=0.04$) |
| *Age at first pregnancy* | | | | | |
| <20 | 86 | 24 | 1[d] | 1[d] | 1[d] |
| 20–24 | 215 | 137 | 0.45 (0.27–0.76) | 0.45 (0.27–0.75) | 0.56 (0.31–1.02) |
| 25–29 | 129 | 138 | 0.26 (0.16–0.42) | 0.28 (0.17–0.47) | 0.47 (0.21–1.04) |
| ⩾30 | 56 | 67 | 0.24 (0.13–0.42) | 0.22 (0.12–0.40) | 0.40 (0.14–1.12) |
| Nulligravidae | 42 | 90 | 0.12 (0.07–0.21) | 0.13 (0.07–0.23) | 0.25 (0.11–0.59) |
| $\chi^2_1$ (trend)[e] | | | 30.93 ($P<0.001$) | 34.49 ($P<0.001$) | 7.05 ($P=0.008$) |
| *Age at first livebirth* | | | | | |
| <20 | 77 | 16 | 1[d] | 1[d] | 1[d] |
| 20–24 | 211 | 134 | 0.36 (0.18–0.56) | 0.33 (0.18–0.59) | 0.42 (0.19–0.92) |
| 25–29 | 125 | 141 | 0.18 (0.10–0.31) | 0.19 (0.11–0.34) | 0.33 (0.13–0.83) |
| ⩾30 | 59 | 60 | 0.22 (0.11–0.41) | 0.19 (0.09–0.36) | 0.48 (0.15–1.15) |
| Nulliparae | 56 | 105 | 0.11 (0.06–0.20) | 0.08 (0.04–0.15) | 0.17 (0.07–0.38) |
| $\chi^2_1$ (trend)[e] | | | 31.32 ($P<0.001$) | 38.01 ($P<0.001$) | 3.07 ($P=0.08$) |
| *Age at last livebirth* | | | | | |
| <25 | 81 | 57 | 1[d] | 1[d] | 1[d] |
| 25–34 | 251 | 216 | 0.80 (0.56–1.20) | 0.87 (0.59–1.28) | 0.83 (0.56–1.22) |
| ⩾35 | 140 | 78 | 1.33 (0.84–2.11) | 1.41 (0.89–2.23) | 1.27 (0.80–2.02) |
| Nulliparae | 56 | 105 | 0.37 (0.23–0.59) | 0.33 (0.20–0.54) | 0.28 (0.17–0.47) |
| $\chi^2_1$ (trend)[e] | | | 2.27 (n.s.) | 2.64 (n.s.) | 1.96 (n.s.) |

[a]Mantel–Haenszel estimates adjusted for age; [b]Mantel–Haenszel estimates adjusted for age and number of sexual partners; [c]Mantel–Haenszel estimates adjusted for age and age at first intercourse; [d]Reference category; [e]Nulligravidae/nulliparae excluded.

The control group for cases with CIN were women with normal cervical smear from the same screening clinics where cases had been identified. A total of 262 controls (median age 37 years, range 18–73) were interviewed. The control groups were not strictly matched with cases for age; however, the age distribution of cases and controls was reasonably well comparable (Table I).

*Data analysis*

We computed the relative risks (RR) of cervical neoplasia, together with their 95% approximate confidence intervals (CI) (Breslow & Day, 1980) according to various reproductive factors, controlling for the potential confounding effects of the major known or potential risk factors for cervical neoplasms using stratification and the Mantel–Haenszel procedure (Mantel & Haenszel, 1959). When a factor could be classified in more than two levels, the significance of the linear trend was assessed by the Mantel test (Mantel, 1963).

In order to allow simultaneously for the effects of several potential confounding factors, we used unconditional multiple logistic regression, fitted by the method of maximum likelihood (Baker & Nelder, 1978). Included in the regression equations were terms for quinquennium of age, marital status (ever married *vs.* never), education (<7; 7–11; ⩾12 years), age at first intercourse (⩽17, 18–22, ⩾23 years), number of sexual partners (0–1, 2, ⩾3), history of Pap smears (never, 1, 2, ⩾3), smoking habits (never, ex-smokers, current smokers <15 and ⩾15 cigarettes per day), oral contraceptive use (never; ⩽2 years; >2 years), plus the reproductive variables still significant after stratified analyses.

**Table III** Multivariate relative risks of invasive cervical cancer according to selected reproductive variables, Milan, Italy, 1981–87

| | *Relative risk estimates (95% CI)* | |
|---|---|---|
| *Variables* | *MLR*[a] | *MLR*[b] |
| *Number of livebirths* | | |
| 0 | 1[c] | – |
| 1 | 2.00 (1.13–3.55) | 1[c] |
| 2 | 2.67 (1.47–4.84) | 1.47 (0.95–2.26) |
| 3 | 4.37 (2.24–8.50) | 2.44 (1.44–4.14) |
| 4 | 3.18 (1.71–8.10) | 2.20 (1.29–4.30) |
| ⩾5 | 4.39 (2.07–9.28) | 2.48 (1.31–4.69) |
| $\chi_1^2$ (trend) | 19.15 ($P$<0.001) | 17.57 ($P$<0.001) |
| *Age at first livebirth* | | |
| <20 | 1[c] | 1[c] |
| 20–24 | 0.51 (0.25–1.05) | 0.61 (0.29–1.27) |
| 25–29 | 0.37 (0.16–0.83) | 0.61 (0.23–1.23) |
| ⩾30 | 0.42 (0.17–1.01) | 0.76 (0.30–1.92) |
| Nulliparae | 0.18 (0.08–0.43) | – |
| $\chi_1^2$ (trend)[d] | 13.15 ($P$<0.001) | 0.02 (n.s.) |
| *Number of induced abortions* | | |
| 0 | 1[c] | 1[c] |
| 1 | 1.39 (0.76–2.55) | 1.30 (0.66–2.48) |
| ⩾2 | 1.26 (0.66–2.40) | 1.30 (0.66–2.60) |
| $\chi_1^2$ (trend) | 0.83 (n.s.) | 0.88 (n.s.) |

[a]Estimates from multiple logistic regression equations including terms for age, marital status, education, age at first intercourse, number of sexual partners, history of Pap smears, smoking habits, oral contraceptive use and one in turn of the variables considered in this table; [b]Estimates from multiple logistic regression equations including the above listed variables, plus number of livebirths and age at first livebirth simultaneously, but excluding nulliparae; [c]Reference category; [d]Nulliparae excluded.

### Results

The distribution of cases and controls according to age, marital status and education is given in Table I. Invasive cervical cancer cases (although not intraepithelial neoplasms) tended to be less frequently unmarried and less educated than the controls.

With reference to nulliparous women, the risk of invasive cervical cancer increased with increasing number of births, being about five times greater in women with five or more births. This trend in risk was statistically significant. No association emerged with number of births with forceps or caesarean births (data not presented), and history of spontaneous abortions (Table II).

Cases of invasive cervical cancer tended more frequently to report induced abortions: the age-adjusted relative risks, compared with no induced abortions, were respectively 1.89 and 2.38 for women reporting one and two or more episodes. Allowance for number of sexual partners did not noticeably modify this association, whereas the risk estimates were somewhat reduced after allowance for age at first intercourse (Table II).

Compared to women whose first pregnancy was before the age of 20 years, the relative risks for those aged 20–24, 25–29 and 30 or more were respectively 0.45, 0.26 and 0.24 ($\chi_1^2$ for trend adjusted for age = 30.93, $P$<0.001). In this case, too, the association was reduced by allowance for age at first intercourse, which led to a reduction of the number of subjects in strata producing meaningful information, since age at first intercourse could obviously not be greater than age at first pregnancy. Similar estimates emerged when the role of age at first livebirth was considered, whereas age at last birth had no apparent effect on the risk of cervical cancer (Table II).

The multivariate relative risks of invasive cervical cancer for number of livebirths, age at first livebirth and number of induced abortions are given in Table III. The estimated values for number of livebirths were broadly consistent with those adjusted for age and number of sexual partners, but the multivariate trends in risk according to age at first livebirth and number of induced abortions were no longer significant after allowance for parity.

The interaction between parity and the other major recognised risk factors for cervical cancer, i.e. indicators of sexual habits, is considered in Table IV. The relative risk increased in subsequent strata of parity with increasing number of sexual partners or decreasing age at first intercourse, and in subsequent strata of partners or age at first intercourse with increasing parity. Thus, the effect of the two factors appeared independent (i.e. multiplicative on the relative risk). Compared with the low parity/low number of sexual partners category, the estimated relative risk for the high parity/high number of sexual partners category was 45.8 (lower 95% confidence limit = 15.3).

Reproductive factors and risk of intraepithelial cervical neoplasia are considered using a scheme similar to that for invasive neoplasm in Table V. No consistent association emerged with parity, number of abortions and age at first pregnancy, first and last birth.

### Discussion

The findings of this study indicate that the number of births has an independent and relevant role in invasive cervical cancer. The risk, in fact, increased markedly with increasing parity and was over four-fold in women with five or more births, compared with nulliparous ones. There was an effect of earlier first birth on cervical cancer risk, too, which could be largely explained in terms of higher parity among women who had their first child earlier. In contrast, no important influence of reproductive variables was observed on pre-invasive cervical neoplasms.

It is unlikely that the association observed between parity and invasive cervical cancer is incidental. The magnitude of the association by itself suggests that it cannot be totally accounted for by bias alone.

**Table IV** Interaction between parity, number of sexual partners and age at first intercourse in the risk of invasive cervical cancer, Milan, Italy, 1981–87

| | Relative risk estimates[a] for number of livebirths (95% CI) | | |
|---|---|---|---|
| | 0 | 1 | ≥2 |
| *Number of sexual partners* | | | |
| ≤1 | 1[b] | 2.50 (1.48–4.21) | 4.33 (2.76–6.79) |
| 2 | 6.50 (2.34–17.93) | 3.35 (1.15–9.77) | 6.29 (3.18–12.43) |
| ≥3 | 4.74 (1.93–11.67) | 15.28 (5.22–44.71) | 45.82 (15.33–139.98) |
| *Age at first intercourse (years)* | | | |
| ≥23 or never | 1[b] | 1.04 (0.57–1.91) | 2.36 (1.44–3.88) |
| 18–22 | 1.85 (0.91–3.77) | 3.06 (1.71–5.46) | 3.69 (2.28–5.97) |
| ≤17 | 2.86 (0.81–10.15) | 7.64 (2.39–24.49) | 7.72 (4.05–14.74) |

[a]Mantel–Haenszel estimates adjusted for age; [b]Reference category.

**Table V** Relative risk of intraepithelial cervical neoplasia according to reproductive variables, Milan, Italy, 1981–87

| Variables | Intraepithelial cancer | Outpatient controls | Relative risk estimates (95% CI) M–H[a] | M–H[b] | M–H[c] |
|---|---|---|---|---|---|
| *Number of livebirths* | | | | | |
| 0 | 67 | 42 | 1[d] | 1[d] | 1[d] |
| 1 | 90 | 62 | 0.86 (0.51–1.44) | 0.96 (0.62–1.72) | 0.91 (0.56–1.53) |
| 2 | 114 | 109 | 0.63 (0.39–1.01) | 0.87 (0.51–1.49) | 0.73 (0.45–1.79) |
| 3 | 38 | 33 | 0.68 (0.37–1.26) | 0.83 (0.41–1.68) | 0.74 (0.39–1.41) |
| ≥4 | 26 | 16 | 0.85 (0.39–1.83) | 0.92 (0.39–2.18) | 0.80 (0.36–1.71) |
| $\chi^2_1$ (trend) | | | 0.93 (n.s.) | 0.16 (n.s.) | 0.89 (n.s.) |
| *Number of spontaneous abortions* | | | | | |
| 0 | 278 | 224 | 1[d] | 1[d] | 1[d] |
| 1 | 43 | 30 | 1.14 (0.69–1.88) | 1.21 (0.73–2.01) | 1.24 (0.75–2.04) |
| ≥2 | 14 | 8 | 0.78 (0.32–1.87) | 1.31 (0.50–3.45) | 1.47 (0.59–3.66) |
| $\chi^2_1$ (trend) | | | 0.56 (n.s.) | 1.36 (n.s.) | 1.27 (n.s.) |
| *Number of induced abortions* | | | | | |
| 0 | 275 | 204 | 1[d] | 1[d] | 1[d] |
| 1 | 42 | 41 | 0.80 (0.50–1.28) | 0.75 (0.46–1.23) | 0.58 (0.33–1.00) |
| ≥2 | 18 | 17 | 0.81 (0.41–1.62) | 0.70 (0.34–1.43) | 0.68 (0.33–1.42) |
| $\chi^2_1$ (trend) | | | 0.90 (n.s.) | 1.36 (n.s.) | 2.28 (n.s.) |
| *Age at first pregnancy* | | | | | |
| <20 | 45 | 21 | 1[d] | 1[d] | 1[d] |
| 20–24 | 125 | 120 | 0.46 (0.26–0.81) | 0.47 (0.25–0.88) | 0.86 (0.40–1.74) |
| ≥25 | 116 | 90 | 0.55 (0.30–1.00) | 0.56 (0.30–1.77) | 1.15 (0.51–2.83) |
| Nulligravidae | 49 | 31 | 0.74 (0.37–1.47) | 0.66 (0.32–1.37) | 1.00 (0.42–2.43) |
| $\chi^2_1$ (trend)[e] | | | 0.48 (n.s.) | 0.63 (n.s.) | 0.55 (n.s.) |
| *Age at first livebirth* | | | | | |
| <20 | 39 | 13 | 1[d] | 1[d] | 1[d] |
| 20–24 | 116 | 113 | 0.37 (0.17–0.62) | 0.33 (0.16–0.70) | 0.52 (0.23–1.21) |
| 25–29 | 84 | 74 | 0.38 (0.19–0.76) | 0.36 (0.18–0.69) | 0.69 (0.27–1.79) |
| ≥30 | 29 | 20 | 0.47 (0.19–1.16) | 0.45 (0.17–1.18) | 0.47 (0.10–2.34) |
| Nulliparae | 67 | 42 | 0.51 (0.24–1.06) | 0.40 (0.18–0.90) | 0.72 (0.29–1.79) |
| $\chi^2_1$ (trend)[e] | | | 1.81 (n.s.) | 1.34 (n.s.) | 1.08 (n.s.) |
| *Age at last birth* | | | | | |
| <25 | 61 | 48 | 1[d] | 1[d] | 1[d] |
| 25–34 | 182 | 150 | 0.94 (0.60–1.47) | 1.05 (0.66–1.69) | 1.42 (0.85–2.38) |
| ≥35 | 25 | 22 | 0.76 (0.37–1.60) | 0.95 (0.43–2.10) | 1.52 (0.58–4.02) |
| Nulliparae | 67 | 42 | 1.01 (0.60–1.72) | 0.91 (0.56–1.52) | 1.25 (0.71–2.22) |
| $\chi^2_1$ (trend)[e] | | | 0.32 (n.s.) | 0.74 (n.s.) | 0.86 (n.s.) |

[a]Mantel–Haenszel estimates adjusted for age; [b]Mantel–Haenszel estimates adjusted for age and number of sexual partners; [c]Mantel–Haenszel estimates adjusted for age and age at first intercourse; [d]Reference category; [e]Nulliparae/nulligravidae excluded.

Selection should not represent a major problem in this study, since cases and controls were identified in institutions covering broadly comparable catchment areas and, despite the rather sensitive nature of the interview, participation was almost complete. Likewise, information bias can hardly have a role on variables like parity or age at first livebirth. In relation to confounding, simultaneous allowance for several potential distorting factors, including measures of social status and indicators of sexual habits, did not appreciably change the parity-related risk.

In this study, no important interaction was observed between reproductive variables and the other major group of factors related to the risk of cervical cancer, i.e. indicators of sexual habits. Thus, the relative risk for women in the highest exposure levels for both groups of factors was grossly elevated, with a point estimate of over 40 for women with two or more births and three or more partners compared with nulliparae with one or no partners. The association observed in this study between parity and invasive cervical cancer agrees with some, but not all, previous investigations. Earlier epidemiological work tended to explain the apparent relation between reproductive variables and cervical cancer in terms of distorting effect by sexual factors (Boyd & Doll, 1964; Rotkin, 1967; Wynder et al.,

1954), but there are at least three studies from India (Jussawalla *et al.*, 1971), Barbados (Barron & Richart, 1971) and the United States (Brinton *et al.*, 1987) which showed important and independent effects of parity and age at first pregnancy or birth on the risk of cervical cancer. It is thus difficult to establish whether the differences between studies are due to bias or to real underlying differences in the populations studied.

Dysplasia of the cervix uteri has been less extensively studied, but the absence of association in this study is consistent with earlier studies from Britain (Harris *et al.*, 1980), Czechoslovakia (Vonka *et al.*, 1984) and Chile (Molina *et al.*, 1988).

There are at least three possible biological interpretations for the association between parity and invasive cervical cancer. First, pregnancy and delivery (and chiefly the first one) (Coppleson & Reid, 1966; Singer, 1975) and possibly mismanaged parturition (Smith, 1931) are a cause of ectropion, which in turn favours the exposure of squamocolumnal junction to carcinogens. Second, pregnancy acting as local immunodepressant can favour infection or the oncogenic potential of viruses such as papilloma virus (Petrucco *et al.*, 1976). Third, the hormonal profile of pregnancy could favour, or accelerate, cervical carcinogenesis, with a mechanism similar to that put forward to explain the risk of cervical neoplasia in long-term oral contraceptive users (Brinton *et al.*, 1987), in terms for instance of glucocorticoid-dependent oncogenic transformation by selected papilloma virus types (Pater *et al.*, 1988). The absence of association with pre-invasive disease in this study – if not due to confounding or other biases introduced, for instance, by the different selection criteria of the two control groups – is similar to the pattern of risk described in relation to oral contraceptive use in the same population (La Vecchia *et al.*, 1986*a*), and therefore suggests a hormone-mediated effect on one of the latter stages of the process of carcinogenesis (Stern *et al.*, 1977), although it is difficult, on this basis alone, to rule out other possible biological mechanisms.

This study was conducted within the framework of the CNR (Italian National Research Council) Applied Project 'Oncology' (contract no. 85.02209.44). The contributions of the Italian League against Tumours and of the Italian Association for Research on Cancer, Milan, Italy, are gratefully acknowledged. The authors wish to thank Dr Mario Sideri for his helpful comments in the preparation of the manuscript and Ms Maria Nigro, Ivana Garimodi and Judy Baggott for editorial assistance.

### References

BAKER, R.J. & NELDER, J.A. (1978). *The GLIM System, Release 3.* Numerical Algorithms Group: Oxford.

BARRON, B.A. & RICHART, R.M. (1971). An epidemiologic study of cervical neoplastic disease. Based on a self-selected sample of 7,000 women in Barbados, West Indies. *Cancer*, **27**, 978.

BOYD, J.T. & DOLL, R. (1964). A study of the aetiology of carcinoma of the cervix uteri. *Br. J. Cancer*, **18**, 419.

BRESLOW, N.E. & DAY, N.E. (1980). *Statistical Methods in Cancer Research, Vol. 1, the Analysis of Case–control Studies.* IARC: Lyon.

BRINTON, L.A. & FRAUMENI, J.F. JR. (1986). Epidemiology of uterine cervical cancer. *J. Chron. Dis.*, **39**, 1051.

BRINTON, L.A., HAMMAN, R.F., HUGGINS, G.R. and 4 others (1987). Sexual and reproductive risk factors for invasive squamous cell cervical cancer. *JNCI*, **79**, 23.

COPPLESON, M. & REID, B. (1966). A colposcopic study of the cervix during pregnancy and puerperium. *J. Obstet. Gynaec. Br. Cwth*, **73**, 575.

HARRIS, R.W.C., BRINTON, L.A., COWDELL, R.H. and 4 others (1980). Characteristics of women with dysplasia or carcinoma *in situ* of the cervix uteri. *Br. J. Cancer*, **42**, 359.

JUSSAWALLA, D.J., DESHPANDE, V.A. & STANDFAST, S.J. (1971). Assessment of risk patterns in cancer of the cervix: a comparison between greater Bombay and Western countries. *Int. J. Cancer*, **7**, 259.

LA VECCHIA, C., DECARLI, A., FASOLI, M. and 5 others (1986*a*). Oral contraceptives and cancers of the breast and of the female genital tract. Interim results from a case–control study. *Br. J. Cancer*, **54**, 311.

LA VECCHIA, C., FRANCESCHI, S., DECARLI, A. and 4 others (1986*b*). Sexual factors, venereal diseases, and the risk of intraepithelial and invasive cervical neoplasia. *Cancer*, **58**, 935.

MANTEL, N. (1963). Chi-square tests with one degree of freedom; extensions of the Mantel–Haenszel procedure. *J. Am. Stat. Assoc.*, **58**, 690.

MANTEL, N. & HAENSZEL, W. (1959). Statistical aspects of the analysis of data from retrospective studies of disease. *JNCI*, **22**, 719.

MOLINA, R., THOMAS, D.B., DABANCENS, A. and 4 others (1988). Oral contraceptives and cervical carcinoma *in situ* in Chile. *Cancer Res.*, **48**, 1011.

PATER, M.M., HUGHES, G.A., HYSLOP, D.E., NAKSHATRI, H. & PATER, A. (1988). Glucocorticoid-dependent oncogenic transformation by type 16 but not type 11 human papilloma virus DNA. *Nature*, **335**, 832.

PETRUCCO, O.M., SEAMACK, R.F., HOLMES, K., FORBES, I.J. & SYMONS, R.G. (1976). Changes in lymphocyte function during pregnancy. *Br. J. Gynaecol.*, **83**, 245.

RIGONI-STERN (1842). Fatti statistici relativi alle malattie cancerose che servirono di base alle poche cose dette dal dott. Rigoni-Stern il dĭ 23 settembre alla Sottosezione di chirurgia del IV Congresso degli scienziati Italiani. *G. Servire Prog. Patol. Ter.*, **2**, 2 (reprinted in *Epidemiol. Prev.*, **3**, 1978).

ROTKIN, I.D. (1967). Adolescent coitus and cervical cancer: associations of related events with increased risk. *Cancer Res.*, **27**, 603.

SINGER, A. (1975). The uterine cervix from adolescence to menopause. *Br. J. Obstet. Gynaecol.*, **82**, 81.

SMITH, F.R. (1931). Etiologic factors in carcinoma of the cervix. *Am. J. Obstet. Gynecol.*, **21**, 18.

STERN, E., FORSYTHE, A.B., YOUKELES, L. & COFFELT, C.F. (1977). Steroid contraceptive use and cervical dysplasia: increased risk of progression. *Science*, **196**, 1460.

VONKA, V., KANKA, J., JELINEK, J. and 11 others (1984). Prospective study on the relationship between cervical neoplasia and herpes simplex type-2 virus. I. Epidemiological characteristics. *Int. J. Cancer*, **33**, 49.

WYNDER, E.L., CORNFIELD, J., SCHROFF, P.D. & DORAISWAMI, K.R. (1954). A study of environmental factors in carcinoma of the cervix. *Am. J. Obstet. Gynecol.*, **68**, 1016.